# Determination of the Microbial and Chemical Loads in Rivers from the Quito Capital Province of Ecuador (Pichincha)—A Preliminary Analysis of Microbial and Chemical Quality of the Main Rivers

**DOI:** 10.3390/ijerph17145048

**Published:** 2020-07-14

**Authors:** Pamela Borja-Serrano, Valeria Ochoa-Herrera, Laurence Maurice, Gabriela Morales, Cristian Quilumbaqui, Eduardo Tejera, António Machado

**Affiliations:** 1 Instituto de Microbiología, Colegio de Ciencias Biológicas y Ambientales (COCIBA), Universidad San Francisco de Quito (USFQ), Diego de Robles y Vía Interoceánica, Campus Cumbayá, Casilla Postal 17-1200-841, Quito 170901, Ecuador; pborjas@usfq.edu.ec (P.B.-S.); vochoa@usfq.edu.ec (V.O.-H.); 2 Colegio de Ciencias e Ingeniería, El Politécnico, Instituto Biósfera, Universidad San Francisco de Quito, Quito 170901, Ecuador; joselyn.morales@univ-tlse3.fr (G.M.); cquilumbaqui@alumni.usfq.edu.ec (C.Q.); 3 Department of Environmental Sciences and Engineering, Gillings School of Global Public Health, University of North Carolina at Chapel Hill, Chapel Hill, NC 27599, USA; 4 Geosciences Environnement Toulouse, CNRS/IRD/CNES/Université Paul Sabatier, 14 avenue Edouard Belin, 31400 Toulouse, France; laurence.maurice@ird.fr; 5 Área de Salud de la Universidad Andina Simón Bolívar, Toledo N22-80, P.O. Box 17-12-569, Quito 170143, Ecuador; 6 Facultad de Ingeniería y Ciencias Aplicadas, Grupo de Bioquimioinformática, Universidad de Las Américas, Quito 170125, Ecuador; eduardo.tejera@udla.edu.ec

**Keywords:** river quality, total coliforms, *Escherichia coli* pathotypes, parasites, physico-chemical parameters, major and trace elements

## Abstract

Contamination of natural water sources is one of the main health problems worldwide, which could be caused by chemicals, metals, or microbial agents. This study aimed to analyze the quality of 18 rivers located in Quito, the capital province of Pichincha, Ecuador, through physico-chemical and microbial parameters. The *E. coli* and total coliforms assessments were performed by a counting procedure in growth media. Polymerase chain reaction (PCR) was realized to detect several microbial genera, as well as *Candida albicans*, two parasites (*Cryptosporidium* and *Giardia* spp.) and *E. coli* pathotypes: enterohemorrhagic *E. coli* (EHEC), enteroaggregative *E. coli* (EAEC), enteroinvasive *E. coli* (EIEC) and enteropathogenic *E. coli* (EPEC). Additionally, physico-chemical parameters and major and trace metals were analyzed in each surface water sample. Our results demonstrated that most of the rivers analyzed do not comply with the microbial, physico-chemical, and metal requirements established by the Ecuadorian legislation. In terms of microbial pollution, the most polluted rivers were Monjas, Machángara, Pisque, and Pita Rivers. Furthermore, three out of four analyzed *E. coli* pathotypes (EIEC, EHEC, and EAEC) were detected in certain rivers, specifically: Monjas River showed the presence of EIEC and EHEC; in the Machángara River, EAEC and EIEC were detected; and finally, EIEC was present in the Guayllabamba River. Several physico-chemical parameters, such as pH, COD_total_, and TSS values, were higher than the Ecuadorian guidelines in 11, 28, and 28% of the rivers, respectively. Regarding heavy metals, Zn, Cu, Ni, Pb, Cd, and Mn surpassed the established values in 94, 89, 61, 22, 22, and 17% of the rivers, respectively. Machangara River was the only one that registered higher Cr concentrations than the national guidelines. The values of Al and Fe were above the recommended values in 83 and 72% of the rivers. Overall, based on the physical-chemical and microbiological parameters the most contaminated rivers were Machángara and Monjas. This study revealed severe contaminations in Ecuadorean Rivers; further studies should evaluate the sources of contamination and their impact on public health.

## 1. Introduction

The discharge of wastes and chemical compounds into rivers is one of the biggest sources of environmental contamination, mainly in developing countries, due to a lack of domestic and industrial wastewater treatment [1,2,3]. The absence of water treatment generates an accumulation of environmental pollutants which could lead to severe public health issues [4]. Pollution in rivers can affect different economical sections, such as agriculture, cattle raising, industrial production, and recreational activities [5,6,7]. The increase in microorganisms and anthropogenic contaminants enhances the risk of pathogen outbreaks, bacterial antibiotic resistance, and public health costs [8,9].

Globally, more than 80% of residual waters are released into the environment without any adequate treatment [10]. It has been reported that, worldwide, around two million people die annually due to water-related diseases [11]. In 2000, Ecuador evidenced more than two thousand cases of diseases associated with water microbial pollution, where most of those cases consisted of diarrhea or dysentery associated with pathogens, such as *Escherichia coli* and others [12,13]. The rates of morbidity and mortality by water contamination are increasing in Ecuador [12].

Of Ecuador’s twenty-four provinces, Pichincha holds significant importance because it is where Quito, the capital city of Ecuador, is located. Pichincha contains a population of approximately 2,576,287, and 86.9% of its inhabitants reside in Quito [14,15]. The domestic and industrial wastes produced in Quito are discharged in four main rivers [16], specifically, in Machángara, Monjas, San Pedro, and Guayllabamba. Around 81% of this contamination is due to domestic wastewater discharge, and the remaining 19% of pollution is attributed to industrial waste [16], such as chemicals and oils. Quito currently has a pilot wastewater treatment plant that is treating less than 3% of the total effluent discharges of Quito city [17]. However, in August 2019, the newly elected mayor, Jorge Yunda, inaugurated the project “Vindobona”, in which they expect to treat almost 99% of the wastewaters that end in the rivers from the capital city [18].

Although the contamination of Pichincha’s rivers is visible nowadays, few studies have been published regarding their microbial and chemical quality [19,20,21]. The last study conducted by the municipal water service of Quito (EPMAPS) revealed that most of the rivers in the south part of the capital exceeded the authorized microbial limits of potable water by 3000% [22]. In 2014, Voloshenko and colleagues found emerging organic pollutants along the San Pedro, Guayllabamba and Esmeraldas Rivers, such as carbamazepine and acesulfame. This study also revealed an increase in pollutant concentrations in the surroundings of Quito [21]. Most water quality studies utilize biological indicators such as *Escherichia coli* and total coliform counts [23,24]. However, other potentially pathogenic microorganisms can be identified in the recollected samples and used as biological indicators, such as *Salmonella*, *Pseudomonas*, *Shigella*, and *Legionella* spp., as well as parasites, *Giardia* and *Cryptosporidium* spp. [25,26,27,28]. Water quality can also be evaluated in terms of physico-chemical properties and parameters [19,29], such as metals, which can reach the hydrosystem through natural or anthropogenic sources. The natural causes for the presence of metals in rivers can be attributed to rock erosion or soil weathering, while the ones for anthropogenic sources include industrial, mining, and agricultural discharges or untreated sewage [5,19,29].

The identification of potentially pathogenic microorganisms and the microbial load evaluation are usually done by microbiological classical methods [30] and even by biological molecular techniques [28]. *Escherichia coli* is known to be a commensal bacterium; nevertheless, some strains can be pathogenic for humans or animals [8] and can be considered as a potential public health risk [1]. Therefore, several studies evaluated specific *E. coli* pathotypes [8,30], such as enterohemorrhagic *E. coli* (EHEC), enteroaggregative *E. coli* (EAEC), enteroinvasive *E. coli* (EIEC), and enteropathogenic *E. coli* (EPEC). *E. coli* pathotype identification is usually done by molecular microbiology methods, such as polymerase chain reaction (PCR) [26,28,31]. On the other hand, the counting of commensal *E. coli* and total coliforms is traditionally done by a classical methodology through a specific culture medium [28,30].

The main goal of the present study was to evaluate the microbial load of 18 different rivers from the capital province of Quito (Pichincha), through *E. coli* and total coliform counting and molecular identification of other potential opportunistic and pathogenic microorganisms (genera or species) using PCR detection. A physico-chemical evaluation was also conducted that included an analysis of major elements and trace metals in the river samples to identify the most polluted rivers and to establish if their microbial load is related to the urban and industrial discharges.

## 2. Methods

### 2.1. Sample Site and Collection

**Figure 1:**
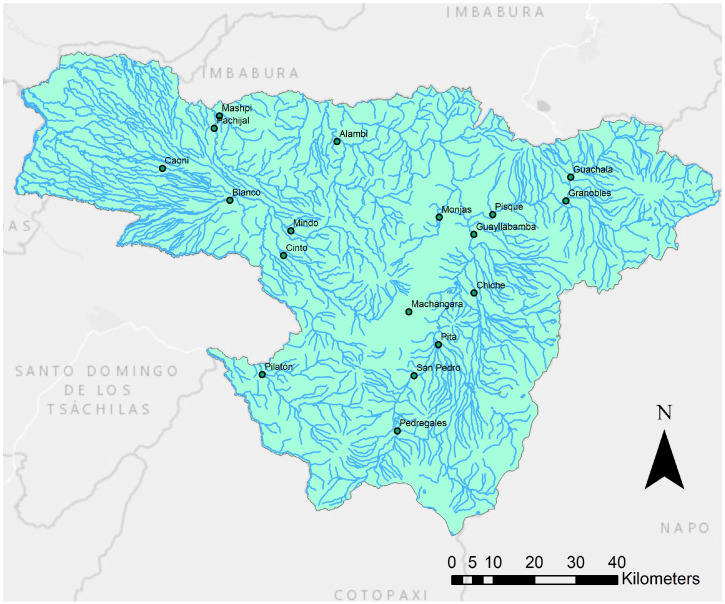
Map of the sample collection points for the 18 rivers (see Supplementary Table S1) from Pichincha province in the present study.

The water samples were collected from 18 rivers located in the province of Pichincha, Ecuador (see Figure 1). It is important to mention that Pichincha is located in the Andean region and is surrounded by coastal and Amazonian regions. This geographical location provides a variety of climates and ecosystems, such as Andean deserts, valleys, and semitropical zones [32]. These rivers were selected because of their location in urban zones and for their accessibility. All samples were collected from urban sites, where the population lived in close contact with the rivers selected for the present study. The samples were collected by fully inverting the sample container and submerging it to a depth of 0.3 m below the water surface, therefore avoiding surface scums and debris [25]. These samples were collected into glass containers previously sterilized by autoclave at 121 °C for 15 min. A total volume of 800 mL was collected for each river, and the samples were maintained at 4 °C until their arrival at the Microbiology Institute at the Universidad San Francisco de Quito. In each river, water samples for the microbial analysis were taken on three different dates of collection (see Table S1).

Additionally, for the physical-chemical analysis, water samples were taken once in each river through: (i) a dark amber glass bottle cleaned in a muffle oven at 550 °C in order to eliminate traces of contaminants and (ii) an acid-clean 1-L Teflon bottle previously washed with 10% HCl at 120 °C and then rinsed with distilled water according to standardized protocols [33]. The samples were conserved at 4 °C until their arrival at the Laboratory of Environmental Engineering at Universidad San Francisco de Quito (LIA-USFQ). The samples were then immediately filtered using a vacuum pump with 0.45-μm pre-cleaned cellulose filters. For metal analysis, the filtrate was transferred to polyethylene bottles and then preserved with high-purity concentrated nitric acid (HNO_3_) (LobaChemie, Mumbai, India) to obtain a final concentration of 2% *v*/*v* at the Laboratory of Environmental Engineering at Universidad San Francisco de Quito (LIA—USFQ), Ecuador.

### 2.2. Sample Preparation for Microbiological Analysis

Surface water samples were filtered using a 0.45-μm nitrocellulose membrane (Millipore, Merck, Burlington, MA, USA) with a vacuum pump under aseptic conditions (Chemical Duty Pump, Milipore, Merck, Burlington, MA, USA). The following procedure was adapted from the study conducted by Dobrowsky and colleagues (2014) with minor modifications. The membrane was removed and placed in a sterile falcon tube with 20 mL of distilled sterile water. The tube was vortexed during 15 min to suspend the soil particles and the microorganisms. Then, the membrane was removed, and the tubes were centrifuged at 5000 rpm for 15 min to precipitate the sediments. Once the pellet was obtained, it was suspended in 2 mL of distilled sterile water. This sample was divided into 2 aliquots of 1 mL, one for bacterial DNA extraction using the Power Soil Extraction Kit (MO BIO Laboratories, QIAGEN, Venlo, The Netherlands) and the other 1 mL for bacterial growth cultures.

### 2.3. Cultivation of Microorganisms from River Samples

Different media cultures were used to isolate or count the microorganisms found in the samples. More accurately, 20 μL of samples were incubated on MacConkey agar (Difco, Becton, Dickinson and Company, Franklin Lakes, NJ, USA) at 37 °C for 18 to 24 h for the recovery of the gener a *Escherichia*, *Salmonella*, *Shigella*, and *Pseudomonas*. Another 20 μL were incubated on Legionella CYE Agar Base (Difco, Becton, Dickinson and Company, Franklin Lakes, NJ, USA) at 35 °C for 48 h for the isolation of *Legionella* spp., and on Biggy agar (Difco, Becton, Dickinson and Company, Franklin Lakes, NJ, USA) to isolate *Candida* spp. Finally, for the quantification of *Escherichia coli* and total coliforms, successive dilutions of the initial aliquot were cultured in Chromocult Agar medium (Merck; Biolab, Wadeville, Gauteng, South Africa) at 35 °C for 24 to 48 h.

### 2.4. DNA Extraction

DNA from the collected water samples was extracted using the instructions for the commercial PowerSoil DNA Isolation Kit (MO BIO Laboratories, Inc., QIAGEN, Venlo, The Netherlands). This commercial kit used PowerBead Tubes, which contained a buffer that dispersed the soil particles and dissolved humic acids and also protected nucleic acids from degradation. The DNA solution from each tube was stored at −20 °C for further PCR analysis.

### 2.5. Molecular Identification of the Microbial Load

#### 2.5.1. Bacterial Genera and Candida Albicans

Once the microbial DNA was extracted from the different samples, 16S conserved rRNA genes were amplified. The PCR mixtures consisted of a final volume of 20 μL and contained 4.0 μL of 1× Green GoTaq Flexi buffer (Promega, Madison, WI, USA), 1.6 μL of 2.0 mM MgCl_2_ (Promega, Madison, WI, USA), 0.4 μL of 0.2 mM dNTPs mix (Promega, Madison, WI, USA), 1.0 μL of each PCR primer (Table 1), 0.2 μL of 0.5 U GoTaq Fexi DNA polymerase (Promega, Madison, WI, USA), 2 μL template DNA and the remaining volume of DNA-free water. For *Shigella* spp., *Salmonella* spp., *Legionella* spp. and *Pseudomonas* spp., the same reaction mixture was used with the exception that 0.09 μL of 0.5 U GoTaq Flexi DNA polymerase were added. Additionally, for the identification of *Candida albicans*, the same reaction mixture was used, with the exception that 2 μL of 2.0 mM MgCl_2_ and 0.18 μL of 0.5 U GoTaq Flexi DNA polymerase were added. The PCR methodology was performed in a thermocycler (Bio-Rad Laboratories, Inc., Hercules, CA, USA) with the procedure illustrated in Table 1.

#### 2.5.2. *Cryptosporidium* and *Giardia* spp.

For the molecular identification of certain well-known parasites, specifically, *Cryptosporidium* and *Giardia* spp., nested PCR was performed using 2 sets of primers for each parasite. The PCR mixtures consisted of a final volume of 25 μL and contained 5 μL of 1× Green GoTaq Flexi buffer (Promega, Madison, WI, USA), 3 μL of 3.0 mM MgCl_2_ (Promega, Madison, WI, USA), 1.0 μL of 0.4 mM dNTPs mix (Promega, Madison, WI, USA), 0.75 μL of each PCR primer (Table 2), 0.07 μL of 0.35U GoTaq Fexi DNA polymerase (Promega, Madison, WI, USA), 1 μL template DNA and the remaining volume of DNA-free water. The nested PCR used the same reaction, with the only difference being that the product of PCR from the pre-nested one was used as template DNA. The PCR methodology was performed in a thermocycler (Bio-Rad Laboratories, Inc., Hercules, CA, USA) with the procedure illustrated in Table 2. 

#### 2.5.3. *Escherichia coli* Pathotypes

For the molecular identification of *E. coli* pathotypes, the PCR mixtures consisted of a final volume of 20 μL. The volume contained 4 μL of 1× Green GoTaq Flexi buffer (Promega, Madison, WI, USA), 1.6 μL of 2.0 mM MgCl_2_ (Promega, Madison, WI, USA), 0.4 μL of 0.2 mM dNTPs mix (Promega, Madison, WI, USA), 0.5 μL of each PCR primer (Table 1), 0.18 μL of 0.5 FU GoTaq Fexi DNA polymerase (Promega, Madison, WI, USA), 2 μL template DNA and the remaining volume of DNA-free water. The PCR methodology was performed in a thermocycler (Bio-Rad Laboratories, Inc., Hercules, CA, USA) with the procedure illustrated in Table 3.

### 2.6. PCR Product Analysis

The PCR products were visualized using electrophoresis with 1.5% agarose gel and staining with ethidium bromide 0.1%, except for *Cryptosporidium* and *Giardia* spp., for which was used a 2% agarose gel. The negative and positive controls used were provided by the Microbiology Institute at Universidad San Francisco de Quito.

#### PCR Product Sequencing

The positive PCR products of *E. coli* pathotypes, *Cryptosporidium* spp., and *Giardia* spp. were then sequenced in Functional Biosciences (Madison, WI, USA) using a Big Dye Terminator V3.1 and run-on ABI 3730xl instruments. Forward and reverse sequence segments were overlapped using PreGap4 and Gap4 (Staden Package, Rodger Staden’s group, Cambridge, England), and the primer sequences were removed. Elucidated nucleotide sequences were compared to the GenBank collection of sequences using the Standard Nucleotide of Basic Local Alignment Search Tool (BLAST). The accuracy of the data was based on the degree of sequence identity.

### 2.7. Analytical Methods

The analysis of physico-chemical parameters was conducted, as described in Benitez et al. [23] and Grube et al. [37], according to the standardized protocols for analysis of water and wastewater [33]. The values of each parameter were obtained by triplicate measurements of each analyzed river sample. Briefly, parameters such as conductivity (SM 2510), pH (SM 4500 H+), temperature and dissolved oxygen (DO) (SM 4500-O A) were measured in situ in triplicates in all the rivers using a portable multiparameter Thermo Scientific Model A329 (Thermo Fisher Scientific, Waltham, MA, USA) (see Figure 1). Turbidity (EPA 180.1 Rev 2.0) was measured with a turbidimeter Thermo Scientific Model AQUAFast AQ4500 (Thermo Fisher Scientific, Waltham, MA, USA). The total chemical oxygen demand (COD_T_) (SM 5520) and phosphates (PO_4_^3−^P) (SM 4500-P B) were measured by colorimetric methods using a Spectronic 20D+ spectrophotometer (Thermo Fisher Scientific, Waltham, MA, USA). Chlorides (Cl^−^) (SM4500 Cl^−^ D), ammonium (NH_4_^+^) (SM 4500-NH_3_), and nitrate (NO_3_^−^) (SM 4500- NO_3_^−^D) were measured using ion-selective electrodes (Thermo Specific Ion Selective Electrode, ISE Orion). In these cases, calibration curves between concentration and potential (mV) (R^2^ = 0.99) were created for each test.

Sulfates (SO_4_^2−^) (SM 426 C) were measured by filtrations using Whatman glass microfiber filters (Grade 934-AH). Total solids (TS) (SM 2540 B) and total suspended solids (TSS) (SM 2540 D) were measured using a 0.45-μm cellulose filter and drying in a 40 GC Lab Oven. The metal analysis was performed on filtered and acidified water samples using a ThermoScientific iCAP 7400 ICP-OES in the Laboratory of Environmental Engineering at Universidad San Francisco de Quito (LIA—USFQ), Ecuador. Calibration curves were created from a multielement standard solution 6 for ICP, grade Trace CERT (Sigma Aldrich, St. Louis, MO, USA), in a concentration of 100 mg/L. The detection and quantification limits were calculated by analyzing blank samples with at least 8 replicates and multiplying the standard deviation by 3 to obtain the limit of detection (LD) and by 10 to obtain the limit of quantification (LQ), respectively.

### 2.8. Quality Assurance/Quality Control

Quality control for major and trace element analysis was conducted by employing certified reference material (CRM 1640a) (NIST, Gaithersburg, MD, USA) every 10 samples (Table S2, see Supplementary Information). The recovery percentages were calculated to determine the matrix effects and to measure the accurateness of the method. All the concentrations of major and trace metals were corrected based on the percentage of recoveries obtained in each analysis, ranging from 89.43% to 105.42%.

### 2.9. Statistical Analysis

The information obtained from the microbial, physico-chemical, and metal analysis of the collected samples was evaluated by using the statistical software package SPSS version 23.0 (IBM Corp, 2013; Armonk, NY, USA). Several linear and multiple regressions were performed between the concentration of *E. coli* and total coliforms, physical-chemical parameters, and major and trace element concentrations. In all the hypothesis tests, a significance level of 5% was used as the standard. In all tests, a *p*-value < 0.05 was considered to be statistically significant.

## 3. Results

### 3.1. Growth of Microbial Genera and Escherichia coli/Total Coliforms Counts

Different media cultures were used to isolate bacteria present in the rivers from this study. In MacConkey agar, all water samples demonstrated growth of enteric bacteria, which included microorganisms form the genera *Escherichia, Salmonella*, *Shigella*, and *Pseudomonas*. As excepted, all rivers showed the presence of enteric bacteria and their further analysis was carried out through PCR (see Section 3.2). During the culture on Legionella CYE Agar Base, 14 out of the 18 rivers presented growth of this genus. Alambí, Blanco, Caoní, and Mindo Rivers did not show any *Legionella* spp. growth on the three water samples recollected. In the Biggy agar, 12 of 18 rivers showed the presence of *Candida* spp. None of the triplicated water samples demonstrated *Candida* growth in the Alambí, Granobles, Guachalá, Monjas, Pisque, or Pita Rivers. These initial results were further evaluated through molecular analysis by PCR, confirming their presence or absence on water samples (see Section 3.2).

The counts of *Escherichia coli* and total coliforms for the 18 rivers evaluated in this study are shown in Table 4. All the analyzed rivers showed concentrations of both *E. coli* and total coliforms that surpassed the maximum allowable concentration, according to the United States Environmental Protection Agency (US EPA), for freshwaters intended for full or partial contact with humans (EPA, 2012), except for the Caoní River on *E. coli* quantification. The highest concentrations of *E. coli* and total coliforms were found in the Monjas, Machángara, and Chiche Rivers, showing levels of *E. coli* and total coliforms between 1.25 × 10^2^–9.18 × 10^2^ and 3.68 × 10^2^–5.15 × 10^3^ CFU/mL, respectively. Moreover, the Caoní and Pilatón Rivers evidenced the lowest values of *E. coli* and total coliforms of the study set, more exactly, 1.17 × 10^0^–1.79 × 10^0^ and 3.95 × 10^0^–4.88 × 10^0^ CFU/mL, respectively.

Using the Ecuadorian legislation [39], comparing our results against the standard values of *E. coli* and total coliforms for quality of water intended for recreational use, it is possible to conclude that only Pilatón and Caoní showed *E. coli* and total coliforms values below the Ecuadorian guidelines, respectively.

### 3.2. Detection of Microbial Genera, Candida Albicans, and Escherichia coli Pathotypes

Molecular analysis was conducted by PCR to confirm the presence or absence of the following microbial genera: *Legionella*, *Pseudomonas*, *Salmonella*, *Shigella*, *Cryptosporidium*, and *Giardia*. Concerning parasites genera, three rivers showed the presence of *Cryptosporidium* spp., which were the following, Mindo, Pisque, and Alambi Rivers. Meanwhile, eight rivers showed the presence of *Giardia* spp., more precisely, the Machángara, San Pedro, Monjas, Blanco, Mindo, Pisque, Pilatón, and Guachalá Rivers. Sequencing and comparison of these products to the GenBank nucleotide collection using BLASTN did not produce any significant alignments for *Cryptosporidium* spp.; however, the positive products for *Giardia* spp. showed 100% homology to *Giardia intestinalis* (syn. *G. duodenalis* or *G. lamblia*). The presence and absence of different bacterial genera, such as *Pseudomonas*, *Salmonella*, *Legionella*, and *Shigella*, were also analyzed in the study set. None of the rivers showed the presence of *Salmonella* spp., while almost all rivers displayed the presence of *Pseudomonas* spp., except the Blanco and Caoní Rivers. The second-most prevalent bacteria genus detected in our study was *Legionella* spp., showing its presence in 11 of the 18 analyzed rivers. Although *Legionella* spp. was detected by growth culture in 14 rivers, *Legionella* species could not be detected in 3 of the 14 rivers through PCR: the Pilatón, Pachijal, and Mashpi Rivers. Finally, three rivers showed the presence of *Shigella* spp., more exactly, the Pita, Monjas and Cinto Rivers. 

The presence of *Candida* species was previously shown in 12 of the 18 rivers through growth culture. However, *Candida albicans* was only detected in three rivers by PCR: the Pita, Monjas, and Blanco Rivers. Furthermore, the detection of four *E. coli* pathotypes was done for all the analyzed rivers: enteroaggregative *E. coli* (EHEC), enteropathogenic *E. coli* (EPEC), enteroinvasive *E. coli* (EIEC) and enteroaggregative *E. coli* (EAEC). Our analysis showed the EIEC pathotype as the most prevalent pathogen in the study set, illustrating positive results in the Machángara, Guayllabamba, and Monjas Rivers. Meanwhile, EHEC and EAEC were each only detected in one river individually, more precisely, the Monjas and Machángara Rivers, respectively. Sequencing and comparing these products to the GenBank nucleotide collection using BLASTN did not produce any significant alignments for these pathotypes due to the low size of the consensus sequences and a great number of gaps. However, positive results were obtained by PCR, as in previous published studies [8,40,41]. Finally, the EPEC pathotype was not detected in any of the 18 rivers evaluated during this study.

### 3.3. Analysis of Physical Parameters and Chemical Elements

Furthermore, we also analyzed the physico-chemical parameters presented in Table 5. The reported values were obtained by triplicate measurements of each analyzed river. These parameters were compared to the maximum contaminant levels (MCL) for the preservation of flora and fauna in fresh water established by Ecuadorean legislation [39]. pH, conductivity, dissolved oxygen (DO), turbidity, redox potential (ORP) and temperature were measured in situ. The remaining physico-chemical parameters were measured at the Laboratory of Environmental Engineering at Universidad San Francisco de Quito (LIA-USFQ). Concerning pH, the Pisque and Machángara Rivers showed the highest pH values above the Ecuadorian threshold, specifically, 9.55 and 9.11, respectively. The minimum pH value registered was 7.15 in both the Chiche and Pachijal Rivers. Conductivity values ranged from 19.87 μS/cm in the Caoní River to 616.00 μS/cm in the Monjas River. According to the US Environment Protection Agency (EPA), the suggested range of conductivity for surface waters is 150–500 μS/cm, meaning that the obtained values in this study are lower and higher than the suggested ones [19]. Half of the analyzed rivers evidenced conductivity values lower than the minimum value (150 μS/cm), more exactly, the Caoní, Mashpi, Pachijal, Chiche, Blanco, Alambi, Pilatón, Mindo and Guachalá Rivers. Additionally, the San Pedro and Monjas Rivers showed higher conductivity values than the maximum limit (500 μS/cm). Turbidity measurements also varied tremendously, between 1.23 NTU in the Blanco River and 881.33 NTU in the Machángara River. Nonetheless, ORP values ranged slightly between 297.13 mV in the San Pedro River to 489.53 mV in the Alambi River. The Monjas River was the only river with a low DO value (5.36 mg/L) and the highest value of DO (10.32 mg/L) was obtained in both the Pachijal and Chiche Rivers. Similarly, regarding the temperature variability, the values ranged from a minimum of 12.40 °C in the Mashpi River to a maximum of 22.30 °C in the Caoní River. Five of the eighteen rivers (28%) presented values of COD_Total_ superior to the Ecuadorian guideline for the preservation of flora and fauna in fresh waters (40 mg/L), more exactly, the Machángara (692 mg/L), Monjas (318 mg/L), Chiche (206 mg/L), Pisque (180 mg/L) and Alambi (65 mg/L). Although all the analyzed rivers showed TS values within the permitted limits for discharges to bodies of water established in the Ecuadorian legislation, five rivers showed TSS values superior to the MCL (130 mg/L), more precisely, the Machángara, Alambi, Chiche, Pisque, and Monjas Rivers, by factors of 4, 2.8, 2.3, 1.8 and 1.2, respectively. Next, the major anion concentrations, such as of chloride, phosphate, sulfate, and nitrate, were within the allowed MCL values for discharges to bodies of water in Ecuador. According to the World Health Organization, the natural level of ammonium in surface waters is usually less than 0.2 mg/L [42]. In our study, ammonium values ranged between 0.13 and 27.48 mg/L, in the Pedregales and Monjas Rivers, respectively. Additionally, the obtained concentration value for fluoride varied from 0.03 to 0.17 mg/L. Hem (1985) reported that, generally, the concentration of fluoride in natural water sources is less than 1.0 mg/L. 

### 3.4. Analysis of Metallic Trace Elements

The concentrations of major and trace metallic elements in the 18 rivers are shown in Table 6. The trace elements analyzed in this study were copper (Cu), cadmium (Cd) chromium (Cr), manganese (Mn), lead (Pb), barium (Ba), nickel (Ni), vanadium (V) and zinc (Zn), while the major elements evaluated were aluminum (Al), iron (Fe), calcium (Ca), sodium (Na), and magnesium (Mg). All metal concentrations were compared to the MCLs for the preservation of flora and fauna in fresh waters established by the Ecuadorian legislation [39]. All rivers registered Cu values above the MCLs, except the Pita and Mashpi Rivers. Four rivers overpassed the MCL for Cd (1 μg/L)—the Machángara, Pilatón, Pedregales, and Guachalá Rivers—by factors of 4, 2, 1.5 and 1.2, respectively. In the case of Cr, just Machángara River exhibited a value, 58.03 μg/L, that surpassed the Ecuadorian MCL (32.0 μg/L). Concerning Mn, the Pedregales, Machángara, and Monjas Rivers showed a slightly higher value than the MCL (10 μg/L). Meanwhile, Pb concentrations were higher than the MCL (1 μg/L) in the Guachalá, Machángara, Pedregales, and Alambi Rivers by factors of 10, 60, 80, and 90, respectively. The concentrations of Ba and V in the 18 analyzed rivers were below the MCL values (1000 μg/L for Ba and 100 μg/L for Va). However, Ni surpassed the MCL value (25 μg/L) in 11 rivers, more exactly, the Pedregales, Guachalá, Machángara, Pisque, Granobles, Pilatón, Chiche, Blanco, Caoní and Cinto Rivers. In the case of Zn, almost all rivers (94%) showed values above the MCL (30 μg/L), with the highest values registered in the Granobles, Monjas, Blanco, Machángara and Pedregales rivers, and the only exception being the Pita River. The values of Fe were above the MCL (0.3 mg/L) in most of the analyzed rivers (72%), in particular for the Alambi, Pisque, Chiche, and Machángara Rivers. Likewise, most of the 18 rivers (78%) showed Al concentrations above the MCL (0.1 mg/L), except the Caoní, San Pedro, Pachijal and Mashpi Rivers, which presented values below the quantification limit. Furthermore, Cd concentrations were higher than the MCL (1 μg/L) in five rivers, more exactly, the Alambi, Guachalá, Pedregales, Pilatón, and Machángara Rivers. Finally, barium and vanadium measures were below the MCL values (1000 and 100 μg/L, respectively) in all rivers.

Unfortunately, some elements, such as Ca, Na, and Mg, are not regulated by Ecuadorian legislation. However, Ecuadorian legislation generally follows US EPA guidelines. The analysis and importance of these major elements are well-known in several studies worldwide. In this study, the Ca levels varied from 3.70 to 170.26 mg/L, in the Caoní and Pedregales Rivers, respectively. Meanwhile, Na concentrations ranged between 4.59 and 73.15 mg/L, in the Caoní and San Pedro Rivers, respectively. Finally, Mg values varied from 2.37 to 32.21 mg/L in the Caoní and San Pedro Rivers, respectively. 

### 3.5. Statistical Analysis

The correlation values between some physico-chemical parameters and the microbial load (*E. coli* and total coliform count) are shown in Table 7. All these physico-chemical parameters showed *p* values below 0.05 against microbial load, meaning that they were statistically significant. According to Mukaka [43], correlation values could be classified into five categories: very high correlations (0.90–1.00), high correlations (0.70–0.90), moderate correlations (0.50–0.70), low correlations (0.30–0.50) and negligible correlations (0.00–0.30). Therefore, the phosphate parameter showed a very high positive correlation within the microbial load. Next, ammonium and sulfate showed a high positive correlation with the microbial load. However, Mn and conductivity only demonstrated a high positive correlation with *E. coli* count, supporting only a moderate positive correlation with total coliform count. Next, Cl^−^ and Na demonstrated a moderate positive correlation with microbial load, while DO revealed a moderate negative correlation with microbial load. Similarly, COD_Total_ showed a moderate positive correlation with *E. coli* count but a low positive correlation against total coliform count. Finally, F^−^ evidenced a low positive correlation with microbial load.

## 4. Discussion

### 4.1. Fecal Coliform Bacteria in River Water Resources

In the current study, most of the rivers showed *E. coli* and total coliform levels above the permitted limits established by the United States Protection Agency [38], except for the Caoní River regarding *E. coli* values. However, according to Texto Unificado Legislación Secundaria del Medio Ambiente (TULSMA) [39], only the Pilatón and Caoní Rivers showed *E. coli* and total coliform values below Ecuadorian regulations. Our results were in agreement with previous studies performed in other Latin American countries. The minimum and maximum values of *E. coli* and total coliform count obtained in our river set and in other previous studies are shown in Table 8. All these studies were conducted in several natural water resources, mainly rivers near or in urban areas.

Furthermore, the *E. coli* levels obtained in the rivers of this study (1.17–9.18 × 10^2^ CFU/mL) were similar to the results reported in Ubá Creek from Brazil [44] and in the Cautín and Imperial Rivers from Chile [45]. However, Carvalho et al. [44] and Rivera et al. [45] reported lower results for total coliforms when compared to the present study. These studies were conducted in similar water resources on the outskirts of the cities Minas Gerais [44] and Nueva Imperial [45]. On the other hand, studies in the Upper Mississippi River in Minnesota from the United States [6] and in three watersheds of the Atlantic region in Canada [46], also located near urban zones, reported lower levels of *E. coli* and total coliforms, respectively, when compared to our study or even to other Latin American countries. As the rivers in our study set, these rivers are also used as drinking water sources and for other activities, such as swimming, bathing, kayaking, and others [6,46]. Studies performed in some countries in Europe, such as Croatia, Italy, and Poland, reported similar levels of *E. coli* [47,48,49], showing much lower contamination levels when compared to the results obtained in the present study (see Table 4). However, countries in Asia (such as India and Malaysia) and Africa (such as Nigeria, Ghana, and Egypt) showed similar levels of *E. coli* when compared to studies in Latin American countries, including this study. A possible explanation for the slight difference in the microbial values measured in Latin America as compared to those in the USA, Canada, and Italy could be the lack of wastewater treatment plants, while the unique climate and biodiversity of Ecuador could also explain higher microbial load in relation to other developing countries such as Ghana and Egypt [50]. It is well known that the proximity of volcanoes surrounding Pichincha province plays an important role in sediments and soil fertility, leading to a richness of microbial load and diversity, as reported in several studies [51,52,53]. Additionally, the precipitation climate of Pichincha province could lead to an increment in microbial load. Previous studies demonstrated that rain events may lead to an inflow of high nutrient concentrations as well as high loads of microbes [54,55]. Fresh water is seasonally plentiful, generally from October through June, with runoff diminishing or fluctuating drastically the rest of the year [15]. Thus, these reasons could explain our results in the present study. However, most countries in Asia and Africa also possessed wastewater treatment plants, which lead to less polluted water sources [56,57]. On the contrary, in Ecuador, most industrial and domestic effluents are directly discharged into rivers, without any previous microbial or physical-chemical treatment. Several industries related to textiles, treated wood and food processing are in the Pichincha area, releasing their effluents with little or no treatment into water resources [15,58,59]. Pichincha province is between two ridges with a series of upland valleys with elevations ranging from 2000 to 3000 m. These upland valleys descend in elevation from north to south, with Quito (Ecuador’s capital) being in the northern Sierra [15]. In 2013, a pilot wastewater treatment plant opened in the Pichincha Province, in the district of Quito, but it could not supply the treatment required for all the rivers analyzed in this study [17] since the wastewater treatment plant located in the Southern area of Quito only treats <3% of the city’s effluent. Nowadays, Quito’s municipal government is working on a project to treat almost 99% of the city’s effluent to reduce the contamination from the wastewaters [18].

The values obtained in the correlation analysis showed that, in this study, there is a strong relationship between the microbial load and certain ions, more exactly, phosphorus, ammonium, and sulfate (see Table 7). Even though these values do not surpass the limits established by Ecuadorian legislation, they could be taken into account to estimate the microbial load in rivers. It is well known that most of the bacterial microorganisms usually conduct fermentation of biological compounds to proliferate and thus produce phosphorus, ammonium, and sulfate as metabolism derivates [60]. Therefore, it could be possible to use these chemical parameters (phosphorus, ammonium, and sulfate) as potential indicators of persistent and elevated microbial loads in river water analysis. Vadde et al. [61] and Vrzel et al. [62] conducted long-term observations of persistent fecal contamination (*E. coli* and total coliforms) in several seasons and spatial locations of the Sava Basin (Slovenia) and Tiaoxi (China) Rivers, respectively, using microbial source tracking (MST) methods through phosphorus (TP), nitrite-N (NO2-N), and ammonium-N (NH4-N) measurements. Therefore, additional studies should be performed in order to conduct further analysis in several seasons and different spatial locations in the Ecuadorian rivers evaluated in this study.

### 4.2. E. coli Pathotypes Detection

The present study showed the presence of three *E. coli* pathotypes in some rivers of the Pichincha province, more specifically, EAEC, EHEC, and EIEC. The most prevalent pathogen was EIEC. showing positive results in 3 out of the 18 rivers, more exactly, the Machángara, Guayllabamba, and Monjas Rivers. EHEC and EAEC were only detected in the Monjas and Machángara Rivers, respectively. When compared to the present study, other countries, such as Australia, South Africa, and Nigeria, reported the presence of four *E. coli* pathotypes [70,71,72]. This diversity in pathotypes could be due to several sanitation issues and zoonotic transmission by wild and livestock animals [73,74,75]. Ramírez Castillo and colleagues [8] identified EAEC as the most prevalent *E. coli* pathotype in the water set of San Pedro River (Aguascalientes State, Mexico), in contrast to our results. EAEC is the second-most common cause of travelers’ diarrhea after ETEC, in both developed and developing countries [76]. EAEC are commonly recognized as a cause of endemic and epidemic diarrhea worldwide and, recently, have been shown to cause acute diarrheal illness in newborns and children in industrialized countries [77]. In the same way, a study performed in Japan reported the presence of EPEC and EAEC in the Yamato River [78], while another study in Germany reported the presence of EIEC and EPEC in low percentages in the Rhine River [79]. EPEC is one of the most important pathogens infecting children less than 2 years of age in the developing world [80], but its prevalence may vary due to differences in study populations, age groups and types of samples or diagnostic methodologies [81]. Lastly, EIEC is a pathotype of *E. coli* that uses the same invasive mechanisms as *Shigella* spp. [82], being one of the leading causes for diarrheal mortality and morbidity [83]. Some studies have described the infectious potential of EIEC to cause food-related public health outbreaks [82,84,85], but limited research has been performed on this subject [82]. Public health regulations apply only to Shigella spp. infections, but they usually lack simple methods to distinguish them from EIEC [82].

It is important to mention that the higher amount of *E. coli* pathotypes usually found in tropical or sub-tropical countries can be attributed to warmer water conditions that facilitate the survival rate of *E. coli* pathotypes [86]. Thus, the presence of different *E. coli* pathotypes in developed countries as compared to developing ones could be attributed to climate variations and environment context, as previously discussed in Section 4.1. This situation could be a threat to public health since the local legislation only focuses on microbial and physical-chemical parameters, while the presence of pathogenic bacteria should also be considered [87]. In addition, most of the rivers receive discharges from several sources, such as agricultural farms, livestock or breeding farms, and also wastewaters from urban and industrial areas. Therefore, it is challenging to establish the main point sources of pollution in the rivers of our study set without further seasonal and spatial analysis of each river. Nonpoint sources of pollution must also be considered in the evaluation of the microbial load in each river. It is important to mention that heavy rain or similar events in tropical countries may increase the number of pathogens in river sediments as well as the presence of contaminants of nonpoint sources (such as fecal material from domestic and wild animals) in the rivers [71,87]. In Quito, the capital city of Ecuador, several rivers are located near agricultural or livestock farms, and they also receive discharge from industrial wastewaters and municipal sewage without previous treatment [16]. Finally, rivers are commonly used for recreational activities, agriculture, livestock feeding, or even domestic activities (such as bathing, washing clothes, and drinking water) [2], leading to severe public health issues, mainly diarrheal-associated diseases [13], with Ecuador not being the exception.

### 4.3. Analysis of Commensal and Parasitic Microorganisms

Furthermore, other bacterial genera were also detected in this study, such as *Pseudomonas* and *Legionella*. These results were not surprising because both genera are known to be abundant and commensal in water resources [25,31]. However, some species of both *Pseudomonas* and *Legionella* genera have been associated with diseases, more exactly, *P. aeruginosa* and *L. pneumophila* [31,87], respectively. Nonetheless, other non-bacterial species have been reported in water sources [87,88,89], such as *Candida albicans* and parasites (*Giardia* and *Cryptosporidium* spp.). *Candida albicans* was detected in a low percentage in our study set, in 3 of the 18 analyzed rivers. Even though *Candida* sp. has been associated with freshwater, this result was expected because this yeast is commonly found in mucocutaneous areas and alimentary tracts of mammals or birds [90]. Cook and Schlitzer [90] revealed that the presence of *Candida albicans* in rivers commonly came from a recent source of contamination by human or animal feces. Other species of *Candida*, such as *C. parapsilosis*, *C. krusei*, *C. glabrata*, and *C. tropicalis*, have also been associated with fresh water [91] and opportunistic infections [34]. *Cryptosporidium* and *Giardia* spp. parasites were also detected in 3 and 8 of the 18 rivers, respectively, evidencing greater parasite contamination in the Pichincha River than with *Candida albicans*. In Germany, a study on the Rhine River showed similar results, isolating a bigger percentage of *Giardia* than *Cryptosporidium* species [27]. Most studies lack parasite detection in water analysis or show low levels of biologic contamination [6,26]. However, it has been shown by several authors that inhibitory compounds (such as humic acids) in river samples can inhibit nested PCR, leading to false results in parasite detection [25,27]. To avoid this, it is recommended to treat the samples with sodium or hypochlorite to reduce the possible effects of inhibition [27]. Another possible methodical troubleshooting issue, mainly in *Cryptosporidium* oocytes, could be the loss of parasite sample by absorption into the recollection recipients or laboratory material and filtration steps [92]. Thus, further studies should be conducted to isolate pathogenic species from these rivers and fully characterize their virulence properties against public health.

### 4.4. Evaluation of Physico-Chemical Parameters in Water Samples

As previously referred to in Table 5, most of the physico-chemical parameters analyzed in this study were below the maximum allowable levels established by the Ecuadorean legislation for the preservation of flora and fauna in fresh waters, cold or warm, marine waters and estuaries or discharge limits to a fresh water body [39]. Nevertheless, certain parameters were outside of the authorized range, more precisely, pH in 11% of the rivers and COD_T,_ and TSS in 28% of the rivers. When compared to other studies, some rivers in other countries of Latin America, such as Chile [45] and Mexico [63], showed pH values within the range of this study (7.00 to 8.50). Meanwhile, in Brazil, Carvalho and Stapelfeldt [44] reported lower pH levels ranging from 5.48 to 7.30 in creek Ubá. However, studies from rivers in North America (the Mississippi, Mersey, Point Wolfe, and Dunk Rivers) showed pH values in a higher range varying from 3.20 to 9.00 [6,46]. The present study showed the highest pH value (9.55) reported in the Pisque River when compared to several studies worldwide, shown in Table 8. Usually, higher pH values in surface waters are associated with carbonate rocks of the geographical region and also with wastewaters from residual municipal or industrial discharge effluents [63,93]. DO values obtained in this study were similar to the ranges analyzed in rivers from other countries. However, several rivers from countries worldwide, such as Italy [48], Bangladesh [65], and Malaysia [66], registered extremely low DO values (see Table 8). These low DO values could be explained by the discharge of untreated wastewaters with high concentrations of organic matter [94]. In Ecuador, when studying water quality parameters in the Machángara River in a longitudinal analysis (DO, biodegradability index (BOD/COD) and total nitrogen), Vizcaíno et al. [20] observed that high temperatures had a negative effect on DO by decreasing its value. Thus, longitudinal studies should be conducted to clarify variables associated with the inconsistency of these physico-chemical parameters.

Five rivers (Alambi, Pisque, Chiche, Monjas, and Machángara Rivers) showed high levels of total chemical oxygen demand (COD_T_). Rivera and colleagues [45] detected similar COD_Total_ levels in Chile’s Cautin and Imperial Rivers. However, in the Atoyac (Mexico) and Ubá (Brazil) Rivers, studies reported greater differences in COD_T_ range, reaching contamination levels of 1841 and 9324 mg/L [44,63], respectively. Regarding other countries worldwide, such as Italy [48], Malaysia [66] and Egypt [69], the obtained values for COD were lower than the ones from this study. Some authors have suggested that these high COD values can be related to the discharge of wastewater and agricultural activities, which normally increase the concentration of organic matter in the river [4].

Finally, the values of total suspended solids (TSS) in our study showed that 28% of rivers registered values above the MCL (Monjas, Pisque, Chiche, Alambi, and Machángara Rivers). Most rivers worldwide registered high levels of TSS, surpassing the maximum permitted limit. Concerning the present study, high levels of TSS (3.33–520.00 mg/L) were reported in the Machángara, Alambi, Chiche, Pisque, and Monjas Rivers. Likewise, rivers from Brazil [44] and Poland [47] showed similar high measures of TSS. It is important to mention that high values of TSS could be associated with several climate and geographical conditions [66], such as recent rainfall, organic or inorganic particles suspended in surface water, and even higher rates of soil erosion produced by human activities. Several examples of these conditions in Pichincha province are given in Section 4.1 and Section 4.2.

### 4.5. Determination of Minor and Major Elements in Water Samples

Minor and major elements were also measured in the water samples of the 18 rivers, as previously shown in Table 6. In the case of the trace metals, the concentrations were higher than those established by Ecuadorian legislation [39] for Zn, Cu, Ni, Pb, Cd, and Mn in 94, 89, 61, 22, 22, and 17% of the rivers analyzed in this study, respectively. Finally, Cr was higher than the recommended value in the Machangara River. In the case of major elements, Al and Fe values were higher than the recommended guidelines in 83 and 72% of rivers analyzed, respectively. As previously mentioned, 89% of the rivers surpassed the MCL values for Cu; this could be explained by the possible presence of organic or inorganic compounds from agricultural pesticides used near those rivers or also by the mineral composition of the soil [95]. A single value of Cr surpassed the guidelines by a factor of 1.6. This peak value was registered in the Machángara River, which could be attributed to discharges from industries that are located near the river [95]. The presence of Cr in water bodies can be related to discharges from the cement, dye, construction, metallurgy, paint (with anticorrosive compounds), and leather industries [96,97]. It is important to mention that several of these industries are located in the Pichincha area. Four rivers in our study set also showed higher values of Cd when compared to MCL: the Machángara, Pilatón, Pedregales, and Guachalá Rivers. According to the WHO [42], high Cd concentrations are related to the presence of steel or plastic industries, which can also be found in the Pichincha area. Additionally, Cd is commonly used in agricultural fertilizers, and, during agricultural runoff, it could reach water sources [98]. High Fe values found in this study are comparable to values reported in other countries. A possible explanation for these high values of Fe could be the discharge of untreated effluents from metallurgical industries located near these rivers, probably similar to the Fe and Al companies previously described next to the Nworie River in Nigeria [67] and metal contaminations already described in other rivers or regions in Ecuador [51,99,100,101]. Alternatively, they could reflect natural sources due to the soil composition [95]. On the other hand, the Pedregales, Machángara, and Monjas Rivers showed higher concentrations of Mn than the allowed value in Ecuadorian legislation [39]. Comparing this result with other countries, Gowrisankar and colleagues [64] reported a low range of Mn levels in the Adyar River and Chembarambakkam Lake in India. Gowrisankar and colleagues also suggested that high concentrations of metals, such as Al, Fe, and Mn, could be attributed to domestic sewage contaminants, for instance, metal scraps, batteries, paints, or oils from service stations [64]. Moreover, this study also showed higher concentrations of Al in comparison with other American countries, such as Mexico [63], Brazil [44], Chile [45], and the USA [6]. However, in Canada, Khan and colleagues [46] showed similar contamination levels of Al in the Mersey, Point Wolfe and Dunk Rivers. Additionally, 22% of the rivers showed high levels of Pb, more exactly, the Machángara, Alambi, Guachalá, and Pedregales Rivers. In 1985, a study demonstrated that rivers located near volcanic zones usually contain higher concentrations of metals, such as Al, Pb, and Fe [95]. In another study performed in Mexico, they found high concentrations of metals such as Fe, Al, and Mn, which they also attributed to the location of the river near a volcanic zone [53]. Since the majority of the rivers located in Pichincha originate on the highlands and are located near volcanoes [32], this could be a plausible explanation for the guideline-surpassing values of Al and Fe metals. Nonetheless, the high concentrations of Al could also be associated with discharge of industrial wastewaters, as already postulated in a previous study [102]. Furthermore, only the Pita River showed a value of Zn within legal limits. Other studies also obtained similar values, more precisely, for the Ubá Creek in Brazil (3.88 mg/L) [44]. While several studies in other Latin American countries (Mexico and Chile), North American countries (USA and Canada), a European country (Italy), Asian countries (India and Bangladesh) and an African country (Ghana) reported lower Zn values in their river analyses, not one was within the MCL established by Ecuadorean legislation. The high Zn concentrations in rivers could be associated with industrial discharges in Ecuador [29] and Brazil [103]. In this study, sixteen of the eighteen rivers showed higher values of copper than the maximum legal value established by Ecuadorian legislation. Some sources mention that Cu can be dispersed to the environment from water pipes from households or industries [95]. Comparing our values of Cu with other studies worldwide, only Mexico evidenced similar concentrations [63]. However, several studies from other countries, such as Chile, the USA, Canada, India, Bangladesh, Nigeria and Ghana, reported higher values of Cu in their rivers analysis. High concentrations of copper could be explained by mining industries or activities near the water sources [103]. They could also be explained due to the geological characteristics of the river [45], while lower concentrations of copper could be explained by adsorption on mineral surfaces [95]. Major elements were also analyzed in this study, revealing a high range of concentrations for Ca, Na and Mg. Although no maximum legal value of these major elements is described in Ecuadorian legislation, this study showed the highest values of Ca when compared to other studies worldwide. Regarding Na, only the Sutla River in Croatia [49] and the Adyar River in India [64] evidenced values higher than in the present study. However, some studies of rivers in India (Adyar River) [64], USA (Mississippi River) [6], and Egypt (Nile River) [69] reported concentrations of Mg almost twice as high as the values reported in this study. These high concentrations of major elements have been described in rivers located near volcanos according to Meybeck and Helmer [93]. It is also important to note that the Pedregales River showed the highest concentration of Ca, and it is also located in an industrial area for dairy products, which are often enriched with this metal [104]. Consequently, industrial discharges could also pollute the river with high levels of Ca through their untreated effluent discharges into the Pedregales River. Additional studies should be conducted to compare control and impacted areas across the river. A longitudinal analysis should be carried out to establish if the presence of metals in surface water samples is attributable to natural or anthropogenic sources.

## 5. Conclusions

In summary, this study revealed diverse and severe contamination in most of the 18 rivers located in the province of the capital city, Quito (Pichincha), in Ecuador. The level of contamination was characterized by different types of parameters, microbial load and several microbial genera, physico-chemical parameters, and metal levels. These 18 rivers are commonly used in rural areas for drinking purposes and recreational, agricultural, and industrial activities. The initial analysis of the microbial parameters in 18 rivers from Pichincha showed high levels of fecal contamination (*E. coli* and total coliforms), the presence of several microbial species (*Pseudomonas* and *Legionella* spp., *Candida albicans*, *Cryptosporidium* and *Giardia* spp.) and also *E. coli* pathotypes (EAEC, EHEC, and EIEC). *Cryptosporidium* and *Giardia* spp. were detected in three and eight rivers, respectively, evidencing greater parasite contamination in the rivers of Pichincha. Sequencing analysis confirmed the positive results for *Giardia* spp., identifying with 100% homology to *Giardia intestinalis*. The Monjas and Machángara Rivers showed the highest number of *E. coli* pathotypes. In both cases, two pathotypes were identified by PCR, more precisely, EHEC and EIEC in the Monjas River and EAEC and EIEC in the Machángara River. The physico-chemical results were higher than the Ecuadorean guidelines for COD_Total_ and TSS in 28% of the rivers evaluated. In the case of the heavy metals, Zn, Cu, Ni, Pb, Cd, and Mn exceeded the guidelines in 94, 89, 61, 22, 22 and 17% of the rivers analyzed in this study. Al and Fe exceeded the guidelines in 83% and 72% of the rivers. Furthermore, both microbial and physico-chemical analysis revealed that the most contaminated rivers were the Machángara and Monjas. It is highly recommended to conduct a spatial analysis in different seasons to evaluate the effects of climate conditions on the microbial and physico-chemical parameters. Finally, different point and non-point sources of pollution in each river, as well as their impact on public health, should also be analyzed.

## Figures and Tables

**Table 1:** Primers and PCR cycling parameters for the detection of various potential bacterial pathogens and *Candida albicans.*

Organism	Primer Name	Primer Sequence (5′–3′)	PCR Cycling Parameters	Gene (Size [bp])	References
Universal	Forward: fDD2	CCGGATCCGTCGACAGAGTTTGATCITGGCTCAG	3 min at 94 °C; 30 cycles of 94 °C for 30 s, 53 °C for 30 s, 72 °C for 1.5 min	16S rRNA (1600)	[25,26]
	Reverse: rPP2	CCAAGCTTCTAGACGGITACCTTGTTACGACTT			
Shigella spp.	Forward: IpaH-F	CCTTGACCGCCTTTCCGATA	2 min at 95 °C; 35 cycles of 94 °C for 1 min, 62 °C for 1 min, 72 °C for 2.5 min, 72 °C for 3 min	*Invasion plasmid antigen H* (606)	[25,26]
	Reverse: IpaH-R	CAGCCACCCTCTGAGGTACT			
Legionella spp.	Forward: JFP	AGGGTTGATAGGTTAAGAGC	5 min at 95 °C; 40 cycles of 94 °C for 1 min, 57 °C for 1.5 min, 72 °C for 1 min, 72 °C for 5 min.	*Attachment invasion locus gene* (386)	[25,26]
	Reverse: JRP	CCAACAGCTAGTTGACATCG			
Salmonella spp.	Forward: IpaB-F	GGACTTTTTAAAAGCGGCGG	2 min at 95 °C; 35 cycles of 94 °C for 1 min, 62 °C for 1 min, 72 °C for 2.5 min, 72 °C for 5 min.	*Invasion plasmid antigen B* (314)	[25,26]
	Reverse: IpaB-R	GCCTCTCCCAGAGCCGTCTGG			
Pseudomonas spp.	Forward: PA-GS-F	GACGGGTGAGTAATGCCTA	2 min at 95 °C; 35 cycles of 94 °C for 20 s, 54 °C for 20 s, 72 °C for 40 s, 72 °C for 5 min	16S rRNA (618)	[25,26]
	Reverse: PA-GS-R	CACTGGTGTTCCTTCCTATA			
Candida albicans	Forward: CALB1	TTTATCAACTTGTCACACCAGA	5 min at 95 °C; 35 cycles of 94 °C for 30 s, 58 °C for 30 s, 72 °C for 30 s, 72 °C for 10 min.	*ITS-1*, *ITS-2* (278)	[34]
	Reverse: CALB2	ATCCCGCCTTACCACTACCG			

**Table 2:** Primers and PCR cycling parameters for the detection of *Cryptosporidium* and *Giardia* spp.

Organism	Primer Name	Primer Sequence (5′–3′)	PCR Cycling Parameters	Gene (Size [bp])	References
*Cryptosporidium* spp.	Forward: Cry 15	GTAGATAATGGAAGAGATTGTG	10 min at 95 °C; 45 cycles of 94 °C for 30 s, 52 °C for 30 s, 72 °C for 50 s.	*COWP* (550)	[35,36]
	Reverse: Cry 9	GGACTGAAATACAGGCATTATCTT			
	Forward: Cowpnest F	TGTGTTCAATCAGACACAGC	10 min at 95 °C; 32 cycles of 94 °C for 30 s, 60 °C for 30 s, 72 °C for 50 s.	*COWP* (311)	
	Reverse: Cowpnest R	TCTGTATATCCTGGTGGG			
*Giardia* spp.	Forward: AL3543	AAATTATGCCTGCTCGTCG	5 min at 94 °C; 35 cycles of 94 °C for 45 s, 50 °C for 45 s, 72 °C for 1 min.	*TPI* (605)	[35]
	Reverse: AL3546	CAAACCTTTTCCGCAAACC			
	Forward: AL3544	CCCTTCATCGGTGGTAACTT	5 min at 94 °C; 35 cycles of 94 °C for 45 s, 55 °C for 30 s, 72 °C for 1 min.	*TPI* (530)	
	Reverse: AL3545	GTGGCCACCACTCCCGTGCC			

**Table 3:** Primers and PCR cycling parameters for the detection of *E. coli* pathotypes according to Ramírez Castillo and colleagues [8].

Organism.	Primer Name	Primer Sequence (5′–3′)	PCR Cycling Parameters	Gene (Size [bp])
*EAEC*	Forward: AggRKs1	GTATACACAAAAGAAGGAAGC	Stage 1, initial denaturing at 95 °C for 2 min; stage 2, denaturing at 95 °C for 1 min, primer annealing at 54 °C for 1 min, and elongation at 72 °C for 1 min; for 30 cycles, and stage 3, final elongation step at 72 °C for 10 min.	*aggR* (254)
	Reverse: AggRkas2	ACAGAATCGTCAGCATCAGC		
*EHEC*	Forward: VTcomU	GAGCGAAATAATTTATATGTG		*stx* (518)
	Reverse: VTcomd	TGATGATGGCAATTCAGTAT		
*EPEC*	Forward: SK1	CCCGAATTCGGCACAAGCATAAGC		*eae* (881)
	Reverse: SK2	CCCGGATCCGTCTCGCCAGTATTCG		
*EIEC*	Forward: IpaIII	GTTCCTTGACCGCCTTTCCGATACCGTC		*ipaH* (619)
	Reverse: IpaIV	GCCGGTCAGCCACCCTCTGAGAGTAC		

**Table 4:** Average and standard deviation values of *Escherichia coli* and total coliforms in the analyzed rivers.

River	*Escherichia Coli* (CFU/ml ± SD) 1.26 CFU Per ml ^a^	Total Coliforms (CFU/ml ± SD) 2.00 CFU Per ml ^b^
Machángara	2.25 × 10^2^ ± 17.67	3.25 × 10^2^ ± 35.35
Guayllabamba	1.25 × 10^2^ ± 35.35	3.13 × 10^2^ ± 88.38
SAN Pedro	9.60 × 10^1^ ± 5.89	2.25 × 10^2^ ± 23.57
Pita	1.00 × 10^2^ ± 82.49	3.50 × 10^2^ ± 29.46
Monjas	9.18 × 10^2^ ± 417.40	5.15 × 10^3^ ± 2474.87
Blanco	1.83 × 10^0^ ± 0.00	4.25 × 10^0^ ± 2.23
Mindo	1.72 × 10^1^ ± 22.8	6.78 × 10^1^ ± 92.50
Cinto	2.98 × 10^1^ ± 40.42	7.30 × 10^1^ ± 97.10
Pisque	1.71 × 10^1^ ± 1.77	4.00 × 10^1^ ± 1.18
Chiche	1.25 × 10^2^ ± 70.71	3.68 × 10^2^ ± 70.71
Pilatón	1.79 × 10^0^ ± 0.29	4.88 × 10^0^ ± 0.17
Pachijal	7.75 × 10^0^ ± 9.07	2.32 × 10^1^ ± 27.28
Alambi	7.08 × 10^0^ ± 1.76	2.58 × 10^1^ ± 2.35
Caoní	1.17 × 10^0^ ± 0.23	3.95 × 10^0^ ± 2.29
Mashpi	2.58 × 10^1^ ± 34.35	7.35 × 10^1^ ± 96.52
Guachalá	1.29 × 10^2^ ± 76.60	2.98 × 10^2^ ± 64.81
Granobles	1.67 × 10^1^ ± 2.36	2.46 × 10^1^ ± 1.77
Pedregales	1.17 × 10^1^ ± 0.00	2.29 × 10^1^ ± 4.12

SD: Standard deviation values. ^a^ The permitted level for surface water partial-body contact (for *Escherichia Coli*) by the United States Environmental Protection Agency [38]. ^b^ The permitted level for surface water partial-body contact (for total coliforms) by the United States Environmental Protection Agency [38].

**Table 5:** Average and standard deviation values of physico-chemical parameters and major anions in 18 rivers of the Pichincha Province (Ecuador).

River MCL	pH 6.5–9 ^a^	Conductivity (μS/cm) N/A	DO (mg/L) N/A	Turbidity (NTU) N/A	ORP (mV) N/A	T (°C) N/A	COD_Total_ (mg/L) 40 ^a^	TS (mg/L) 1600 ^b^	TSS (mg/L) 130 ^b^	Cl^−^ (mg/L) 1000 ^b^	NH_4_^+^N (mg/L) N/A	NO_3_^−^N (mg/L) 13 ^a^	PO_4_^3−^P (mg/L) 10 ^b^	SO_4_^−^ (mg/L) 1000 ^b^	Fluoride (mg/L) 1.0 ^b^
Machángara	9.11 * ± 0.03	297.97 ± 1.38	6.77 ± 0.24	881.33 ± 12.66	362.70 ± 3.61	15.20 ± 0.30	692.00 * ± 6.13	1359.00 ± 4.24	520.00 * ± 18.86	37.27 ± 1.04	20.36 ± 0.87	6.40 ± 0.07	0.17 ± 0.01	29.00 ± 0.00	0.14 ± 0.00
Guayllabamba	7.90 ± 0.03	365.00 ± 5.81	7.42 ± 0.23	56.50 ± 0.66	402.23 ± 0.15	18.20 ± 0.00	33.00 2.27	397.00 ± 7.07	90.00 ± 9.43	26.51 ± 2.01	2.54 ± 0.29	5.13 ± 0.03	1.17 ± 0.01	11.50 ± 0.50	0.03 ± 0.00
San Pedro	8.00 ± 0.01	529.77 ± 0.06	8.23 ± 0.20	22.17 ± 3.30	297.13 ± 3.45	13.43 ± 0.06	20.00 ± 2.36	470.00 ± 14.14	52.00 8.49	23.78 ± 0.54	7.16 ± 0.18	6.95 ± 0.00	1.19 ± 0.14	65.85 ± 6.59	0.17 ± 0.00
Pita	8.41 ± 0.01	221.80 ± 0.00	8.10 ± 0.05	10.73 ± 0.76	346.70 ± 1.55	13.80 ± 0.10	8.00 ± 4.71	280.00 ± 14.14	45.00 ± 28.28	4.45 ± 0.51	0.23 ± 0.05	1.93 ± 0.00	0.50 ± 0.19	71.62 ± 4.12	0.13 ± 0.00
Monjas	8.04 ± 0.05	616.00 ± 0.10	5.36 ± 0.03	136.00 ± 15.10	323.17 ± 0.55	19.60 ± 0.10	318.00 * ± 0.00	632.50 ± 10.61	153.50 * ± 4.95	40.32 ± 1.44	27.48 ± 1.47	3.43 ± 0.00	3.93 ± 0.56	103.72 ± 9.88	0.15 ± 0.00
Blanco	7.32 ± 0.09	53.53 ± 0.08	8.76 ± 0.22	1.23 ± 0.03	310.00 ± 10.41	20.97 ± 0.06	20.00 ± 2.12	470.00 ± 14.14	6.67 ± 4.71	1.11 ± 0.23	4.19 ± 1.54	0.63 ± 0.03	0.05 ± 0.01	3.50 ± 0.50	0.04 ± 0.00
Mindo	8.37 ± 0.16	139.67 ± 0.15	8.27 ± 0.26	1.76 ± 0.11	323.70 ± 0.53	17.87 ± 0.15	2.00 ± 2.12	280.00 ± 14.14	8.33 ± 2.36	9.31 ± 0.04	0.19 ± 0.01	0.70 ± 0.00	0.11 ± 0.01	6.00 ± 0.00	0.04 ± 0.00
Cinto	7.20 ± 0.01	232.93 ± 0.64	8.06 ± 0.18	5.34 ± 0.15	306.00 ± 0.95	20.37 ± 0.29	2.00 ± 2.12	632.00 ± 10.61	6.67 ± 0.00	21.39 ± 0.07	0.39 ± 0.01	0.57 ± 0.00	0.05 ± 0.01	29.00 ± 0.00	0.05 ± 0.01
Pisque	9.55 * ± 0.17	273.43 ± 0.40	8.02 ± 0.08	306.67 ± 4.62	408.20 ± 2.18	16.63 ± 0.12	180.00 * ± 1.53	806.00 ± 28.28	236.67 * ± 33.00	14.04 ± 0.73	0.27 ± 0.02	10.98 ± 0.09	0.11 ± 0.00	6.00 ± 0.00	0.14 ± 0.01
Chiche	7.15 ± 0.01	44.80 ± 0.02	10.32 ± 0.31	5.89 ± 0.21	412.23 ± 11.52	21.40 ± 0.00	206.00 * ± 4.59	597.00 ± 24.04	300.00 * ± 0.00	28.17 ± 1.37	1.01 ± 0.01	6.31 ± 0.09	0.18 ± 0.01	3.50 ± 0.50	0.15 ± 0.00
Pilatón	8.15 ± 0.01	101.67 ± 0.12	8.77 ± 0.20	56.10 ± 3.12	372.23 ± 1.31	17.23 ± 0.06	2.16 ± 2.12	182.00 ± 14.14	54.00 ± 14.14	3.93 ± 0.00	0.22 ± 0.01	0.95 ± 0.03	0.12 ± 0.01	11.00 ± 0.00	0.05 ± 0.00
Pachijal	7.15 ± 0.01	44.80 ± 0.02	10.32 ± 0.31	5.89 ± 0.21	412.23 ± 11.52	21.40 ± 0.00	2.00 ± 0.00	61.00 ± 9.90	3.33 ± 0.00	1.24 ± 0.03	0.22 ± 0.02	0.86 ± 0.03	0.11 ± 0.01	2.00 ± 0.00	0.03 ± 0.00
Alambi	8.15 ± 0.14	72.07 ± 0.12	8.92 ± 0.17	251.33 ± 11.50	489.53 ± 1.12	18.50 ± 0.00	65.00 * ± 2.27	521.00 ± 1.41	366.67 * ± 0.00	3.42 ± 0.15	0.24 ± 0.05	1.25 ± 0.03	0.21 ± 0.02	3.00 ± 0.00	0.04 ± 0.00
Caoní	7.33 ± 0.15	19.87 ± 0.04	9.35 ± 0.33	25.93 ± 1.99	397.07 ± 9.02	22.30 ± 0.00	7.00 ± 2.27	45.00 ± 7.07	20.00 ± 7.07	2.31 ± 0.00	0.21 ± 0.04	11.66 ± 0.06	0.09 ± 0.02	3.50 ± 0.50	0.05 ± 0.00
Mashpi	8.15 ± 0.01	33.72 ± 0.12	9.87 ± 0.50	11.07 ± 1.01	435.40 ± 3.65	N/A	9.00 ± 0.00	36.00 ± 11.31	8.33 ± 7.07	1.06 ± 0.04	0.22 ± 0.03	1.19 ± 0.00	0.06 ± 0.00	4.00 ± 0.00	0.03 ± 0.00
Guachalá	8.11 ± 0.02	147.00 ± 0.69	7.78 ± 0.62	7.60 ± 0.27	381.40 ± 0.00	12.40 ± 0.00	2.00 ± 0.00	407.50 ± 10.61	21.67 ± 2.36	2.53 ± 0.03	0.29 ± 0.02	2.60 ± 0.00	0.27 ± 0.01	14.00 ± 0.00	0.07 ± 0.00
Granobles	7.78 ± 0.00	159.00 ± 0.15	6.91 ± 0.07	16.70 ± 0.46	424.23 ± 0.93	13.80 ± 0.00	13.00 ± 2.27	182.50 ± 10.61	28.33 ± 2.36	4.69 ± 0.37	0.29 ± 0.01	4.97 ± 0.06	0.59 ± 0.01	6.50 ± 0.50	0.04 ± 0.00
Pedregales	7.67 ± 0.26	194.00 ± 0.61	6.72 ± 0.08	11.60 ± 0.26	328.83 ± 0.64	13.53 ± 0.23	2.00 ± 0.00	222.00 ± 5.66	18.33 ± 2.36	13.26 ± 0.64	0.13 ± 0.00	1.56 ± 0.09	0.30 ± 0.01	6.00 ± 0.00	0.06 ± 0.00

^a^Table 2: Quality criteria acceptable for the preservation of flora and fauna in fresh waters, cold or warm, and marine waters and estuaries. Texto Unificado Legislación Secundaria del Medio Ambiente (TULSMA), Book VI, Annex I [39]. ^b^ Table 9: Discharge limits to a fresh water body. TULSMA, Book VI, Annex I [39]. * Values that exceed the quality criteria. N/A: not available. The reported values were obtained by triplicate measurements of each analyzed river sample.

**Table 6:** Average and standard deviation values of major and trace metallic elements in 18 rivers of the Pichincha Province (Quito, Ecuador).

River	Trace Elements									Major Elements				
MCL	Copper (µg/L) 5 ^a^	Lead (µg/L) 1 ^a^	Chromium (µg/L) 32 ^a^	Manganese (µg/L) 100 ^a^	Barium (µg/L) 1000 ^a^	Cadmium (µg/L) 1 ª	Nickel (µg/L) 25 ^a^	Vanadium (µg/L) 100 ^b^	Zinc (µg/L) 30 ^a^	Aluminium (mg/L) 0.1 ^a^	Iron (mg/L) 0.3 ^a^	Calcium (mg/L) N/A	Sodium (mg/L) N/A	Magnesium (mg/L) N/A
Machángara	38.95 * ± 0.00	59.7 ± 0.00	58.03 ± 0.00	165.52 ± 0.00	541.88 ± 0.00	4.17 * ± 0.22	54.92 * ± 0.00	50.76 ± 0.00	437.37 * ± 0.00	18.05 * ± 0.00	5.39 * ± 0.00	21.2 ± 1.65	31.76 ± 1.03	6.05 ± 0.07
Guayllabamba	10.17 * ± 0.00	<LQ	2.86 ± 0.00	75.50 ± 0.00	340.90 ± 0.00	<LQ	17.92 ± 0.00	28.49 ± 0.00	104.84 * ± 0.00	0.49 * ± 0.00	0.46 * ± 0.00	17.86 ± 2.13	30.71 ± 1.43	13.33 ± 0.43
San Pedro	8.57 * ± 0.00	<LQ	1.64 ± 0.00	48.95 ± 0.00	773.21 ± 0.00	<LQ	<LQ	56.89 ± 0.00	53.44 * ± 0.00	0.03 ± 0.00	0.28 ± 0.00	29.32 ± 2.79	73.15 ± 1.26	34.21 ± 2.87
Pita	<LQ	<LQ	<LQ	44.02 ± 0.00	38.45 ± 0.00	<LQ	<LQ	21.80 ± 0.00	5.22 ± 0.00	0.16 * ± 0.00	0.31 * ± 0.00	16.07 ± 1.52	17.73 ± 0.55	10.73 ± 0.43
Monjas	10.65 * ± 0.00	<LQ	2.27 ± 0.00	208.13 ± 0.00	256.91 ± 0.00	<LQ	3.77 ± 0.00	17.80 ± 0.00	149.67 * ± 0.00	0.18 * ± 0.00	0.26 ± 0.00	24.09 ± 2.97	58.19 ± 1.90	9.28 ± 0.63
Blanco	15.23 * ± 0.00	<LQ	35.18 ± 0.00	10.13 ± 0.00	477.14 ± 0.00	<LQ	36.86 * ± 0.00	18.51 ± 0.00	181.80 * ± 0.00	5.07 * ± 0.00	0.84 * ± 0.00	7.92 ± 0.34	8.46 ± 0.99	2.72 ± 0.27
Mindo	15.68 * ± 0.00	<LQ	36.26 ± 0.00	13.86 ± 0.00	440.11 ± 0.00	<LQ	37.31 * ± 0.00	26.23 ± 0.00	76.77 * ± 0.00	17.66 * ± 0.00	0.43 * ± 0.00	15.96 ± 0.94	12.01 ± 0.40	4.91 ± 0.09
Cinto	10.76 * ± 0.00	<LQ	36.25 ± 0.00	69.92 ± 0.00	427.05 ± 0.00	<LQ	35.33 * ± 0.00	27.15 ± 0.00	68.75 * ± 0.00	17.30 * ± 0.00	0.57 * ± 0.00	17.74 ± 1.75	16.76 ± 0.78	9.00 ± 0.53
Pisque	23.11 * ± 0.00	<LQ	41.58 ± 0.00	27.89 ± 0.00	389.50 ± 0.00	<LQ	42.78 * ± 0.00	47.23 ± 0.00	83.62 * ± 0.00	17.53 * ± 0.00	2.37 * ± 0.00	46.16 ± 3.71	28.62 ± 1.92	12.39 ± 0.78
Chiche	18.07 * ± 0.00	<LQ	41.78 ± 0.00	30.13 ± 0.00	388.95 ± 0.00	<LQ	37.09 * ± 0.00	46.02 ± 0.00	87.70 * ± 0.00	18.08 * ± 0.00	3.95 * ± 0.00	12.71 ± 1.94	20.15 ± 0.74	7.08 ± 0.84
Pilatón	14.88 * ± 0.00	<LQ	42.83 ± 0.00	23.31 ± 0.00	308.75 ± 0.00	2.31 * ± 0.01	39.96 * ± 0.00	26.02 ± 0.00	101.74 * ± 0.00	13.12 * ± 0.00	0.59 * ± 0.00	11.67 ± 1.73	8.78 ± 0.76	4.42 ± 0.49
Pachijal	7.89 * ± 0.00	<LQ	2.03 ± 0.00	1.72 ± 0.00	229.62 ± 0.00	<LQ	<LQ	2.43 ± 0.00	76.32 * ± 0.00	<LOQ	0.04 ± 0.00	5.82 ± 0.01	4.82 ± 0.06	3.28 ± 0.22
Alambi	8.82 * ± 0.00	88.9 ± 0.00	3.01 ± 0.00	41.32 ± 0.00	348.04 ± 0.00	1.02 ± 0.00	3.15 ± 0.00	9.16 ± 0.00	103.90 * ± 0.00	2.06 * ± 0.00	1.15 * ± 0.00	10.98 ± 0.00	8.90 ± 1.32	4.40 ± 0.19
Caoní	5.39 * ± 0.00	<LQ	2.07 ± 0.00	1.13 ± 0.00	253.81 ± 0.00	<LQ	35.33 * ± 0.00	<LQ	58.55 * ± 0.00	0.08 ± 0.00	0.11 ± 0.00	3.70 ± 0.08	4.59 ± 0.21	2.37 ± 0.08
Mashpi	<LQ	<LQ	1.84 ± 0.00	4.11 ± 0.00	82.75 ± 0.00	<LQ	<LQ	<LQ	75.86 * ± 0.00	<LQ	0.02 ± 0.00	5.82 ± 0.08	4.99 ± 0.16	3.22 ± 0.01
Guachalá	12.28 * ± 0.00	12.4 ± 0.00	42.63 ± 0.00	22.26 ± 0.00	248.71 ± 0.00	1.16 * ± 0.03	89.94 * ± 0.00	28.48 ± 0.00	98.91 * ± 0.00	18.25 * ± 0.00	0.88 * ± 0.00	15.23 ± 2.03	14.57 ± 0.94	6.66 ± 0.86
Granobles	17.61 * ± 0.00	<LQ	44.60 ± 0.00	54.99 ± 0.00	389.02 ± 0.00	<LQ	41.37 * ± 0.00	33.65 ± 0.00	146.93 * ± 0.00	18.12 * ± 0.00	0.93 * ± 0.00	13.77 ± 0.81	14.61 ± 0.42	6.63 ± 0.54
Pedregales	11.53 * ± 0.00	86.0 ± 0.00	4.49 ± 0.00	140 * ± 0.00	333.81 ± 0.00	1.49 * ± 0.17	100.11 * ± 0.00	24.50 ± 0.00	3711.6 * ± 168.48	0.39 * ± 0.00	0.87 * ± 0.00	170.26 ± 2.51	17.81 ± 1.02	10.62 ± 0.35

^a^Table 2: Quality criteria acceptable for the preservation of flora and fauna in fresh waters, cold or warm, and marine waters and estuaries. Texto Unificado Legislación Secundaria del Medio Ambiente (TULSMA), Book VI, Annex I [39]. ^b^Table 3: Quality criteria for water for agricultural irrigation. TULSMA, Book VI, Annex I [39]. * Values that exceed the quality criteria. <LQ: below the limit of quantification. N/A: not available. The reported values were obtained by triplicate measurements of each analyzed river sample.

**Table 7:** Correlation values between physico-chemical parameters and microbial load and their correlation categories according to Mukaka [40].

Parameters	*E. coli* (CFU/mL)	Total Coliforms (CFU/mL)	Correlation Category (for *E. coli*/Total Coliforms)
Conductivity (µS/cm)	0.702	0.649	High/Moderate
DO (mg/L)	−0.599	−0.555	Negligible/Negligible
COD_Total_ (mg/L)	0.506	0.376	Moderate/Low
Cl^−^ (mg/L)	0.674	0.578	Moderate/Moderate
NH_4_^+^N (mg/L)	0.870	0.801	High/High
PO_4_^3−^P (mg/L)	0.924	0.938	Very high/Very high
SO_4_^2−^ (mg/L)	0.770	0.726	High/High
Manganese (mg/L)	0.742	0.675	High/Moderate
Fluoride (mg/L)	0.499	0.402	Low/Low
Sodium (mg/L)	0.607	0.547	Moderate/Moderate

Legend: All of these correlations showed a previously significant *p*-value equal or below 0.05.

**Table 8:** Comparison of the physical parameters, chemical elements, and coliform counts obtained in our study and other urban areas around the world.

N°	Country	Study Group (*n*)	Counting		Physico-Chemical Parameters				Major and Trace Elements						References
			*E. coli* (CFU/mL)	Total Coliforms (CFU/mL)	pH	DO (mg/L)	COD_T_ (mg/L)	TSS (mg/L)	Iron (mg/L)	Aluminum (mg/L)	Zinc (μg/L)	Copper (μg/L)	Sodium (mg/L)	Magnesium (mg/L)	
1	Ecuador	18	1.17– 9.18 × 10^2^	3.95– 5.15 × 10^3^	7.15–9.55	5.36– 10.32	2.0–692.0	3.33– 520.00	0.21– 13.88	0.03– 18.05	5.22– 3712	5.39– 38.95	4.59– 58.19	2.37– 32.21	This study
2	Brazil *	1	4.20– 2.40 × 10^2^	4.60 × 10– 2.40 × 10^2^	5.48–7.30	0.90– 7.80	<10.0– 9324.0	56.00– 608.00	0.03– 24.09	0.10– 0.37	30.00– 3880	NA	NA	NA	[44]
3	Chile *	2	2.00 × 10^−2^– 7.90	1.70– 5.40 × 10	7.00–8.50	8.0–12.70	2.0– 406.0	10.87– 260.00	0.21– 0.56	0.002– 0.06	20.00– 140.00	170.00– 630.00	1.60– 8.43	0.95– 1.69	[45]
4	Mexico *	1	2.20 × 10– 3.08 × 10^5^	NA	7.00–8.00	1.70– 8.60	22.0– 1841.0	8.00– 343.00	0.51– 0.53	<5.0	<100.00	<50.00	NA	NA	[63]
5	USA	1	5.00 × 10^−2^– 3.00	NA	6.89–8.10	NA	NA	13.13– 139.42	0.04– 1.59	0.08– 1.18	20.00– 210.00	10.00– 570.00	1.63– 21.39	3.63– 55.54	[6]
6	Canada	3	NA	3.70 × 10– 7.40 × 10^2^	3.20–9.00	9.20– 14.70	NA	NA	0.01– 4.20	0.00– 21.00	0.00– 1000.00	1.00– 110.00	0.30– 17.30	NA	[46]
7	Poland	5	1.58– 1.18 × 10^2^	3.80– 2.98 × 10^2^	7.40–7.70	NA	NA	223.00– 518.00	0.08– 4.40	NA	NA	NA	4.00– 33.50	0.80– 5.40	[47]
8	Italy	4	3.00 × 10^−2^– 4.10 × 10^2^	0– 1.30 × 10^2^	6.90–8.80	1.70– 18.40	4.0– 87.0	4.00– 64632.00	0.001– 0.053	0.0003– 0.28	0.10– 441.00	1.00– 16.30	NA	NA	[48]
9	Croatia *	3	1.00 × 10^−1^– 2.97 × 10^2^	1.01 × 10– 6.67 × 10^3^	7.82–8.24	NA	NA	NA	0.02– 0.52	0.01– 0.07	NA	0.10– 1.27	2.12– 88.30	9.30– 27.10	[49]
10	India	2	3.16 × 10^2^– 7.94 × 10^2^	6.30 × 10^2^– 6.31 × 10^6^	7.10–8.00	NA	NA	172.00– 1820.00	0.25– 0.53	0.00– 0.27	48.60– 102.00	0.00– 406.6	18.00– 406.00	8.00– 55.00	[64]
11	Bangladesh	1	NA	NA	7.24–7.61	1.22– 3.66	NA	239.00– 1349.00	1.40– 3.29	NA	80.00– 190.00	50.00– 100.00	NA	NA	[65]
12	Malaysia	1	4.33– 2.73 × 10^3^	NA	5.23–8.41	4.13– 7.44	8.6– 63.0	17.66– 80.00	NA	NA	NA	NA	NA	NA	[66]
13	Nigeria	1	2.50 × 10– 6.40 × 10^2^	NR– 1.60 × 10^2^	6.84–7.20	NA	NA	8.63– 11.36	2.97– 4.80	NA	NA	6.00– 130.00	2.77– 4.10	6.06– 8.66	[67]
14	Ghana	1	3.36– 7.39	1.13 × 10– 1.88 × 10	7.20–7.48	6.60– 7. 16	NA	142.00– 225.00	0.61– 1.19	NA	14.00– 100.00	28.00– 274.00	NA	NA	[68]
15	Egypt*	1	3.79– 7.03	7.13– 1.42 × 10	7.60– 8.70	NA	4.9– 21.2	NA	NA	NA	NA	NA	NA	32.00– 56.00	[69]

NA: Not analyzed in the study. NR: Not reported in the study. * All these studies used NPM/mL for counting. According to Ecuadorean legislation, the limit for *E. coli* and total coliforms for recreational water use is 200 MPN/100mL and 1000 MPN/100mL, respectively. Table 6: Quality criteria acceptable for recreational water use related to primary contact activities. TULSMA, Book VI, Annex I [39].

## Supplementary Materials

The following are available online at https://www.mdpi.com/1660-4601/17/14/5048/s1, Table S1: Name of the rivers and location on the map of Pichincha, with its coordinates and water samples collection date. Table S2: Recovery percentage, reproducibility percentage, accuracy percentage, detection, and quantification limits obtained from metal analysis employing the ICP-OES.
