# Supplementary material for: Determination of the Microbial and Chemical Loads in Rivers from the Quito Capital Province of Ecuador (Pichincha)—A Preliminary Analysis of Microbial and Chemical Quality of the Main Rivers
Source: Int J Environ Res Public Health. 2020 Jul 14;17(14):5048. doi: 10.3390/ijerph17145048 (PMC7400137; doi:10.3390/ijerph17145048)
Supplement: Supplementary file 1 [file ijerph-17-05048-s001.pdf]

Supplementary Materials:

Table S1. Name of the rivers and location on the map of Pichincha, with its coordinates and water samples collection date.

Location	Rivers	Coordinates	Dates of collection
1	Machángara	0°14'2''S 78°30'54''W	31/01/2016-18/06/16
2	Guayllabamba	0°04'01.8''S 78°22'27.3''W	18/05/2016-31/05/16
3	San Pedro	0°22'17.7''S 78°30'13.1''W	27/01/2017-18/11/17
4	Pita	0°18'16.3"S 78°27'03.6"W	27/01/2017-18/11/17
5	Monjas	0°01'48.5"S 78°26'57.4"W	27/01/2017-13/04/17
6	Blanco	0°00'23.7''N 78°54'12.6''W	10/02/2017-21/01/18
7	Mindo	0°03'33.4''S 78°46'16.2''W	10/02/2017-21/01/18
8	Cinto	0°06'46.2"S 78°47'13.1"W	10/02/2017-21/01/18
9	Pisque	0°01'27''S 78°20'0''W	03/03/2017-10/01/18
10	Chiche	0°11'36.3''S 78°22'25.1''W	03/03/2017-18/11/17
11	Pilatón	0°22'9''S 78°49'60''W	17/03/2017-13/01/18
12	Pachijal	0°09'41.9''N 78°56'14.9''W	24/03/2017-26/01/18
13	Alambi	0°07'59''N 78°40'16''W	24/03/2017-13/04/17
14	Caoní	0°04'31''N 79°02'60''W	24/03/2017-26/01/18
15	Mashpi	0°11'18.5''N 78°55'35.1''W	24/03/2017-06/01/18
16	Guachalá	0°0'19''N 78°10'28''W	07/04/2017-03/12/17
17	Granobles	0°3'22''N 78°9'50''W	07/04/2017-10/01/18
18	Pedregales	0°29'26''S 78°32'25''W	07/04/2017-07/01/18

Table S2. Recovery percentage, reproducibility percentage, accuracy percentage, detection, and quantification limits obtained from metal analysis employing the ICP-OES.

Elements Parameters		Al	Ba	Cd	Cr	Cu	Fe	Pb	Mn	Ni	V	Zn	Co	Ca	Mg	Na
**Recovery (%)**	Run 1	90.67	95.40	96.19	97.24	96.72	93.07	105.81	96.67	89.43	101.41	94.75	-	105.42	93.43	100.38
	Run 2	-	-	112.08	105.21	101.70	-	-	93.27	103.97	100.53	100.92	98.98	91.81	118.53	96.04
**Limit of detection (LD) (µg/L)**	Run 1	1.92	4.09	0.16	0.29	1.23	0.78	3.24	0.18	1.00	2.03	0.42	-	0.20	0.06	0.04
	Run 2	-	-	0.24	0.40	2.50	-	-	0.25	0.83	0.63	0.65	0.51	0.02	0.06	0.09
**Limit of quantification (LQ) (µg/L)**	Run 1	6.39	13.62	0.52	0.97	4.11	2.61	10.81	0.60	3.34	6.77	1.40	-	0.66	0.20	0.14
	Run 2	-	-	0.80	1.33	8.32	-	-	0.83	2.77	2.09	2.16	1.71	0.06	0.021	0.31
**Accuracy (%)**	Run 1	0.19	2.95	4.89	3.55	1.61	0.34	13.09	2.44	2.55	6.75	2.58	-	0.07	2.97	0.78
	Run 2	-	-	3.92	4.12	4.91	-	-	4.72	4.08	5.47	3.87	4.58	0.00	5.30	5.73
	Average	0.19	2.95	4.40	3.83	3.26	0.34	13.09	3.58	3.32	6.11	3.23	4.58	0.03	4.13	3.25
	σ	-	-	0.69	0.40	2.34	-	-	1.61	1.08	0.90	0.91	-	0.03	1.16	2.47
**Reproducibility**	Run 1	0.09	0.05	0.04	0.03	0.03	0.07	0.06	0.03	0.11	0.01	0.05	-	0.05	-0.07	0.00
	Run 2	-	-	0.12	0.05	0.02	-	-	0.07	0.04	0.01	0.01	0.01	-0.08	0.19	-0.04
	Average	0.09	0.05	0.08	0.04	0.02	0.07	0.06	0.05	0.07	0.01	0.03	0.01	-0.01	0.06	-0.02
	σ	-	-	0.06	0.02	0.01	-	-	0.02	0.05	0.01	0.03	-	0.06	0.13	0.02
**CRM 1640a (µg/L)**		53.00	151.80	3.99	40.54	85.75	36.80	12.10	40.39	25.32	15.05	55.64	20.34	5.61	1.05	3.13

Legend: Values obtained with recovery percentages too far from 100% were replaced with (-) sign.
